# Supplementary material for: TastepepAI: An artificial intelligence platform for taste peptide de novo design
Source: PLoS Comput Biol. 2025 Oct 16;21(10):e1013602. doi: 10.1371/journal.pcbi.1013602 (PMC12543283; doi:10.1371/journal.pcbi.1013602)
Supplement: S1 File — (ZIP) [file pcbi.1013602.s006.zip › Supplementary Data 2/TaPep30 HPLC.doc]

**HPLC REPORT**

| Sample: | FCl-NJP93333 TaPep30 AW-5 | | | Analyzed date: | 2024-09-22 |
| --- | --- | --- | --- | --- | --- |
| Analyst: | LLR Reconstitution:1mg/1mlH_2_O | | | | |
| Lot. No.: | P240913-MX1172248 | | | | |
| Column: | Kromasil C18, 4.6*250mm, 5μm | | | | |
| Solvent A | A: 0.1% Trifluoroacetic Acid in 100% Acetonitrile | | | | |
| Solvent B | B: 0.1% Trifluoroacetic Acid in 100% Water | | | | |
| Gradient: |  | A | B |  | |
|  | 0.0min | 16% | 84% |  | |
|  | 25.0min | 41% | 59% |  | |
|  | 25.1min | 100% | 0% |  | |
|  | 30.0min | Stop | |  | |
| Volume: | 20μl | | |  | |
| Wavelength: | 220nm | | |  | |
| Flow rate: | 1.0ml/min | | |  | |
|  | | | | | |
| ────────────────────────────────  Rank Time Area Conc.  ────────────────────────────────  1 10.352 72616 1.981  2 10.450 3482660 95.04  3 11.017 109304 2.983  ────────────────────────────────  Total 3664580 100 | | | | | |
